# A bibliometric study of research pertaining to the oldest-old (age eighty-five and older)

**DOI:** 10.5195/jmla.2020.762

**Published:** 2020-01-01

**Authors:** Brady Daniel Lund, Ting Wang

**Affiliations:** Doctoral (PhD) Student, School of Library and Information Management, Emporia State University, Emporia, KS, blund2@g.emporia.edu; Emporia State University, Emporia, KS, twang2@emporia.edu

## Abstract

**Objective:**

This bibliometric study investigated literature pertaining to a quickly growing population worldwide: the oldest-old, individuals age eighty-five and older. The current state of research was surveyed, based on top authors, publishers, authorship networks, themes in publication titles and abstracts, and highly cited publications.

**Methods:**

Bibliographic data was abstracted from the Web of Science database. Microsoft Excel was used for data analyses related to top author, publishers, and terms. VosViewer bibliographic visualization software was used to identify authorship networks.

**Results:**

Publications pertaining to the oldest-old have increased dramatically over the past three decades. The majority of these publications are related to medical or genetics topics. Citations for these publications remain relatively low but may be expected to grow in coming years, based on the publication behavior about and increasing prominence of this population. Claudio Franceschi and the *Journal of the American Geriatrics Society* were found to be the author and journal with the most publications pertaining to the oldest-old, respectively.

**Conclusions:**

The oldest-old is a population of rapidly growing significance. Researchers in library and information science, gerontology, and beyond can benefit themselves and those they serve by participating in research and specialized services to marginalized populations like the oldest-old. This bibliometric study hopefully serves as a launch-point for further inquiry and research in the years to come.

## INTRODUCTION

A variety of terms exist to describe the population that is the focus of this bibliometric study: very old, oldest-old, and fourth age. Regardless of the term chosen, it is used to describe individuals eighty-five years of age or older. This population is generally retired (with most individuals being retired for ten years or more), experiencing a general decline in physical and mental ability and health (which may result in residence in a nursing or assisted-care facility), and characterized by reflection on life and preparation for the final stage of life: death [1]. This description paints a rather morose outlook for the lifestyles of these individuals; however, many individuals who are age eighty-five and older still live fulfilling lives. While most of the literature on the oldest-old pertains to health and decline, there is a gap in the literature about the everyday activities and behaviors of the oldest-old, particularly among those who are satisfied with life (as opposed to those suffering from depression, anxiety, or dementia).

The oldest-old is a rapidly growing population, expected to double in size by the year 2050 [2]. The average life expectancy in the United States today is seventy-nine years [3]. For those who make it to the age of eighty, the predicted life expectancy is another eight to ten years and for those who make it to ninety, another four to five years [4]. Individuals are no longer reaching the oldest-old stage and immediately passing away; they may reach this stage and still live for another decade or more. As this population grows in size, it is likely that their health, social, and information needs will become a greater priority in research across disciplines.

An early publication on the topic of the oldest-old, which both defined the term and guided subsequent research, is Suzman, Willis, and Manton’s 1992 book *The Oldest Old* [5]. This book is divided into sections that cover demographics, research challenges, health problems, social lives, and policies related to the oldest-old. This publication built upon prior work of Suzman and Manton [6–9] and was likely a crucial part of establishing the formal study of this population. For instance, across the 3 years preceding and following the publication of this book, the number of publications pertaining to the oldest-old jumped by over 100%. Subsequent to this book, several publications have referenced or discussed the body of literature pertaining to the oldest-old, but few with the same thoroughness necessary to examine the entire body of literature. Thus, a new overview of the structure of this research is warranted.

Many prior bibliometric studies have been conducted for topics related to aging and the elderly. Of these studies, which were published between 1991 and 2019, sixteen pertained to physical health and aging, seven pertained to mental health and aging, three pertained to finances, three pertained to social life, and six pertained to other topics (supplemental Appendix A). However, none of these studies examined literature specifically pertaining to the growing population of the oldest-old.

As the focus on the oldest-old as an important and growing population in research intensifies, a bibliometric study of the existing body of oldest-old research is needed. This study was designed to examine trends in journal articles pertaining to research on the oldest-old in terms of major authors and journals, coauthorship networks and relationships, number of publications per year, and top terms or themes emerging from article titles and abstracts. The aim of this research was to characterize publishing patterns of oldest-old research, which can help developmental psychology, gerontology, and library and information science researchers identify seminal sources and topics for future research pertaining to the oldest-old.

## METHODS

The methods for this study were informed by Moed, Glanzel, and Schmoch’s *Handbook of Quantitative Science and Technology Research* [10], particularly the chapter pertaining to coauthorship analysis [11]. Bibliographic data (i.e., authors, title, abstract, text, references) for 5,291 research articles published from 1991 to 2019 pertaining to the oldest-old were collected from Web of Science (WoS) for analysis. The authors selected the year 1991 as a cut-off for selection of articles because this was the year prior to Suzman, Willis, and Manton’s seminal publication *The Oldest Old* and the first year that more than 10 research articles were published on the topic of the oldest-old. WoS was selected based on its popular use in bibliometric studies and its availability at our university. The queried databases included Social Science Citation Index (SSCI), Emerging Sources Citation Index (ESCI), Conference Proceedings Citation Index, and MEDLINE.

All research articles in all languages included in the WoS databases were included in the selection of articles. The search terms used to identify articles were “oldest-old,” “centenarian,” “age 85+,” and “very old.” Searching was performed on March 25–27, 2019. The search results were carefully vetted to ensure that entries were relevant to the topic of this study. For example, searching the topic of “very old” retrieved non-relevant articles describing non-human animal experiments, geology, or primary populations below 80 years of age. After identifying non-relevant articles, we removed their bibliographic information from the data set, reducing the search results to 4,437 articles that were relevant to our research question.

For basic descriptive findings (e.g., top authors or journals, number of publications per year, word frequency), data were exported to an Excel spreadsheet and analyzed using Excel functions. Before analyzing authorship and coauthorship networks, author disambiguation was performed. Word frequency analysis was performed by exporting the title and abstract fields of the Excel file to NVivo and running its frequency analysis feature. For more complex mappings of coauthorship networks, we used VosViewer (version 1.6.11), which is a free software package produced by the University of Leiden that is capable of identifying and visualizing meaningful relationships in bibliographic data imported from WoS. There was no threshold used for the minimum number of documents or citations received by an author for inclusion in this visualization.

## RESULTS

### Authorship of oldest-old research

The 27 authors (top 25 positions, with multiple ties) of oldest-old research with the most publications are displayed in Table 1. No author/anonymous is also listed, bringing the total number of entries on the list to 28. The authors mainly worked in the fields of epidemiology, cardiology, ontology, community and environmental sociology, and geriatric psychology. All but 6 of these authors worked in a medical field. All authors had worked in some postdoctoral professional capacity (e.g., professor or researcher-clinician) for over 10 years, based on information from their curricula vitae (CVs) or professional websites. The country affiliations of the authors with the most publications were the United States (n=12), the People’s Republic of China (n=4), Italy (n=3), Spain (n=2), Brazil (n=1), Denmark (n=1), France (n=1), Germany (n=1), Israel (n=1), and Japan (n=1), and no author/anonymous (n=1). Together, these 28 authors contributed to 299 publications.

**Table 1:** Authors of oldest-old research with the most publications (1991–2019)

Rank	Author	Number of articles
1	Claudio Franceschi	19
2	Thomas T. Perls	18
3	Francesc Formiga	16
3	Yasumichi Arai	16
5	Yi Zeng	15
6	Peter Martin	14
7	(No Author/Anonymous)	13
7	Giuseppe Paolisso	13
9	Jinmyoung Cho	12
10	Fredric D. Wolinsky	11
10	Jean-Marie Robine	11
12	Leonid A. Gavrilov	10
12	Kenneth G. Manton	10
14	Colleen L. Johnson	9
14	Daniela Brandao	9
14	Mette Sorensen	9
14	Yong-Han He	9
14	Truls Ostbye	9
14	Paola Sebastiani	9
20	Katie E. Cherry	8
20	Danan Gu	8
20	Giovanni Ravaglia	8
20	Janine Stein	8
24	Qiukui Hao	7
24	David Leibowitz	7
24	Assumpta Ferrer	7
24	Dellara F. Terry	7
24	Gil Atzmon	7

**Figure 1:**
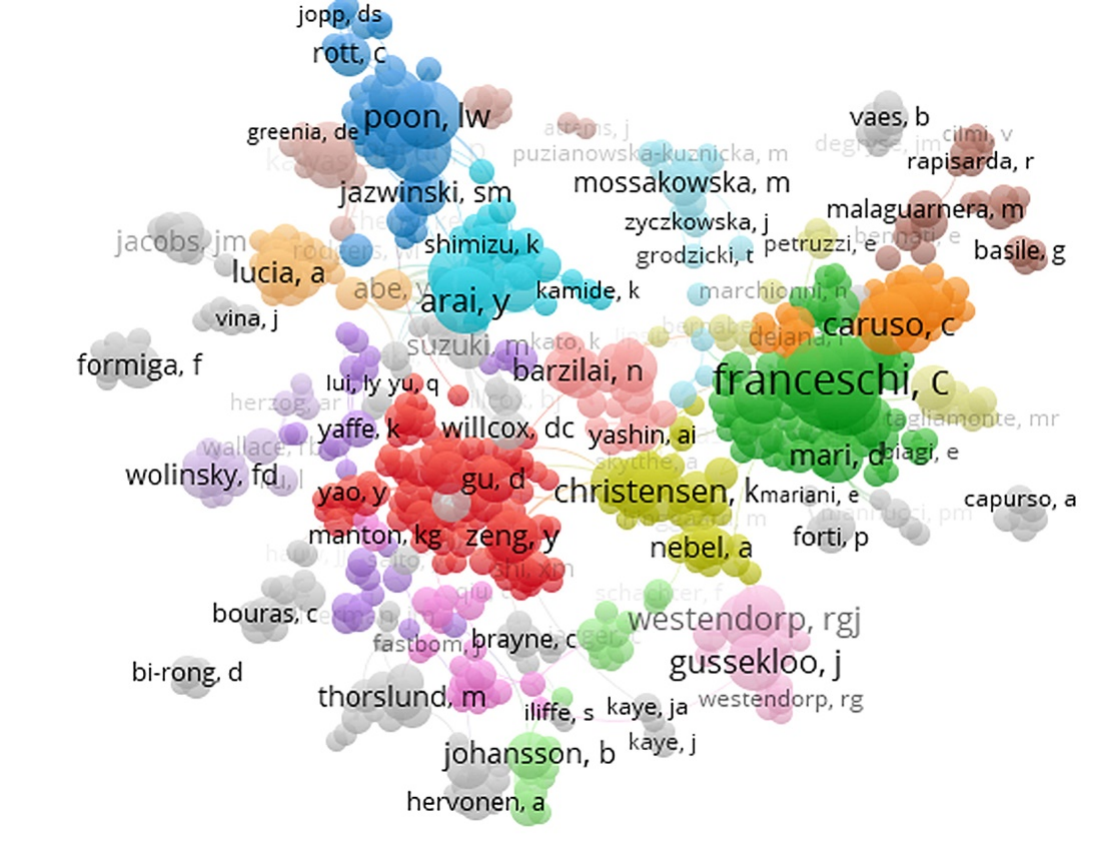
Coauthorship networks in oldest-old research

Figure 1 shows the largest coauthorship network among authors of articles pertaining to the oldest-old. The network is observed to center around Claudio Franceschi, who had 98 direct coauthors, 100 second-degree coauthors (i.e., authors who coauthored an article with an author who coauthored an article with Franceschi), 92 third-degree coauthors (i.e., authors who coauthored an article with an author who coauthored a different article with an author who coauthored another different article with Franceschi), and dozens of distant “relatives.”

Each circle in the visualization represents an author. The size of each circle in the visualization represents the number of publications relative to those of other authors. The shorter the distance between two circles, the closer the relationship between the two authors. That is, authors who are very close together have likely published an article together, whereas authors farther away may only be related through shared coauthors but have never published an article together (as is the case with the second- and third-degree coauthors of Franceschi mentioned above). Each color in the visualization indicates a subnetwork or cluster (n=29) that centers on a different group of authors who have published multiple articles together. The names visible on the clusters are those of authors with the greatest number of publications about the oldest-old (i.e., the largest circles). Overall, this visualization indicates strong interconnections among oldest-old researchers, with large groups of researchers who publish together on the topic, many of whom are connected to the highly influential oldest-old researcher, Franceschi.

### Journals for oldest-old research

Table 2 lists the top 25 journals for oldest-old research published between 1991 and 2019. Most of these journals specifically publish gerontology research, with *PLoS One* and *Neurology* being exceptions. These top journals published 1,548 publications, or 35% of the total oldest-old publications.

**Table 2:** Top journals for oldest-old research (1991–2019)

Rank	Journal	Number of articles
1	*Journal of the American Geriatrics Society*	183
2	*The Journals of Gerontology, Series A*	147
3	*Archives of Gerontology and Geriatrics*	114
4	*Experimental Gerontology*	105
5	*The Journals of Gerontology, Series B*	88
6	*Mechanisms of Aging and Development*	85
7	*Age and Aging*	62
8	*International Journal of Aging and Human Development*	55
9	*Aging and Mental Health*	52
9	*Aging Clinical and Experimental Research*	52
11	*BMC Geriatrics*	51
12	*Gerontology*	49
13	*Nihon Ronen Igakkai Zasshi*	48
14	*PLoS One*	47
15	*Age*	44
16	*Journal of Aging and Health*	43
17	*The Gerontologist*	42
18	*Neurology*	41
19	*Geriatrics and Gerontology International*	39
20	*The Journal of Nutrition, Health and Aging*	38
21	*Zeitschrift für Gerontologie und Geratrie*	34
22	*Neurobiology of Aging*	33
22	*International Psychogeriatrics*	33
24	*International Journal of Geriatric Psychiatry*	32
25	*Journal of the American Medical Directors Association*	31

### Word frequency analysis of oldest-old article titles and abstracts

Word frequencies for oldest-old research article titles and abstracts are displayed in Table 3. Most of these terms were expected based on the study population, with “aging,” “old,” “oldest,” “centenarian,” and “geriatric” making up nearly one-third of content words used in titles and abstracts. Nine of the terms relate to health or medical topics, while “communication,” “association,” and “American” stand out as terms that seem unique from the other terms on the list.

**Table 3:** Term frequency in oldest-old article titles and abstracts (1991–2019)

Rank	Term group	Frequency
1	Aging	5,346
2	Old	3,829
3	Centenarian	2,186
4	Oldest	2,176
5	Functioning	1,728
6	Geriatric	1,489
7	Health	1,480
8	Association	1,478
9	Mortality	958
10	Longevity	923
11	Care	864
12	Disease	781
13	Living	763
14	Cognition	725
15	American	691
16	Genetics	648
17	Patient	618
18	Clinic	543
19	Adult	518
20	Communication	489

### Temporal trends in oldest-old research

**Figure 2:**
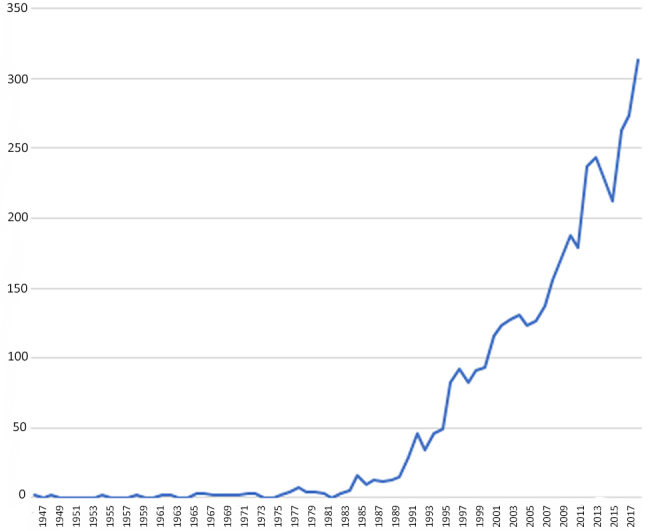
Number of oldest-old research articles by year

Figure 2 shows the number of oldest-old research publications produced each year between 1945 and 2018, based on expanded search criteria in WoS to provide a more complete picture of the history of this research. This analysis shows that research focusing on the oldest-old was virtually nonexistent until the mid-1970s, when it experienced a small emergence. In the mid-1980s, oldest-old research accelerated and, with a few brief exceptions, increased rapidly into the present. From 1987 to 2004, the total number of publications per year has increased by an average of nine publications per year and seventeen per year since 2005.

### Highly cited oldest-old research

Supplemental Appendix B lists the research articles about the oldest-old with at least 100 citations according to data from WoS. These 62 articles represented the top ~2% of oldest-old articles by number of citations. The most cited article, “Inflamm-Aging: An Evolutionary Perspective on Immunosenescence” [12], received 1,589 citations, and the primary author was the most prolific oldest-old research author, Claudio Franceschi. The next 4 most highly cited articles received between 500 and 1,000 citations, including Baltes and Smith’s “New Frontiers in the Future of Aging: From Successful Aging of the Young Old to the Dilemmas of the Fourth Age,” a seminal work that defined the stages of aging, including the fourth age [13]. Additionally, 31 articles had more than 150 citations, and 11 articles had between 250 and 500 citations.

Table 4 shows all authors and journals with multiple publications with at least 100 citations. This table indicates that the most impactful (i.e., receiving large numbers of citations) sources of information about the oldest-old come from a relatively small group of authors (n=5) and journals (n=9) compared with the total number of unique authors (n=2,042) and journals (n=679) publishing at least 1 article on the topic of the oldest-old.

**Table 4:** Authors and journals with multiple articles with more than 100 citations (1991–2019)

	Total number of articles	Articles with 100+ citations	

		n	(%)
Author			
Franceschi	19	4	(21%)
Atzmon	7	3	(43%)
Perls	18	2	(11%)
Longo	2	2	(100%)
Corrada	6	2	(33%)
Journal			
*Journal of American Geriatrics Society*	183	5	(3%)
*Neurology*	41	5	(12%)
*Gerontology Series A*	147	4	(3%)
*Annals of the New York Academy of Science*	24	3	(13%)
*Proceedings of the National Academy of Science*	11	3	(27%)
*Gerontology Series B*	88	3	(3%)
*Gerontology*	49	2	(4%)
*Aging and Mental Health*	52	2	(4%)
*Experimental Gerontology*	105	2	(2%)

## DISCUSSION

There are several potential implications of these findings. First, medical aspects of life for the oldest-old are by far the most prevalent in the published literature. This finding is supported by most top authors (both in terms of number of publications and number of citations) being medical researchers, the top journals being associated with medical disciplines, and the top terms in article titles and abstracts including “health,” “mortality,” “care,” “disease,” and “cognition.”

The field of oldest-old research remains quite young, and its publications generally do not receive large numbers of citations compared to, for instance, “gerontology” as a subject, which has 115 articles with more than 1,000 citations according to WoS. However, this may change in coming years, as the size of the oldest-old population is growing alongside increasing research on this population. The number of publications per year has steadily increased for over 3 decades. This number likely demonstrates the effect of a sustained increase in human longevity and quality of life across the globe, resulting in a recognition that the younger-old (sixty-five to seventy-five years, who may still be working or are recently retired and are often still in good health and more connected with the world around them) and the oldest-old (eighty-five+) may have very different interests and needs, including with regard to health, communication, and information use.

Lastly, some sources of research on the oldest-old tend to have greater influence. Three seminal gerontology journals—*Journal of the American Gerontological Society*, *Gerontology Series A*, and *Experimental Gerontology*, as well as the non-specialized medical journal *Proceedings of the National Academy of Science*—were identified as having a large number of articles pertaining to the oldest-old, many highly cited (100+ citations) articles, or both. For those researching the oldest-old, these may be ideal sources for seeking publication.

The findings of this study extend prior bibliometric studies of gerontology, particularly recent studies such as those by Dominko and Verbic [14], Gu et al. [15], and Shen, Nguyen, and Hsu [16]. These studies, all published in 2019, examined publishing trends among gerontology topics irrespective of a specific population of older adults: well-being among older adults [14], health in aging [15], and general trends in gerontology research [16]. Given that there are significant physiological, neurological, communication, and social divisions between the younger-old and oldest-old [17, 18] as well as divisions in research involving these populations, evaluating gerontological research as though the elderly are a homogeneous population, as was done in prior bibliometric studies, may be problematic. For example, Shen, Nguyen, and Hsu found “dementia” and “Alzheimer’s” to be two of the most frequently occurring words in general gerontological research, but these words do not appear in the top words for articles involving the oldest-old population [16]. It may be worthwhile to reevaluate these studies based on the demographics of the populations in the body of research studied.

Researchers in library and information science, gerontology, and other disciplines can benefit themselves and those they serve by engaging in research that involves this growing population. Librarians may offer specialized services to marginalized populations such as the oldest-old by being informed about the growing importance of this population in academic and medical discourse. New researchers in this field could develop a foundation for their research based on major themes, most published authors, and most cited publications. Thus, this bibliometric study serves as a launch-point for further inquiry and research in the years to come.

## SUPPLEMENTAL FILES

Appendix A Bibliometric studies related to aging and the elderly (1991–2019)

Appendix B Articles with more than 100 citations (1991–2019)

## 

**Brady Daniel Lund**, blund2@g.emporia.edu, Doctoral (PhD) Student, School of Library and Information Management, Emporia State University, Emporia, KS

**Ting Wang**, twang2@emporia.edu, Emporia State University, Emporia, KS
