# Supplementary material for: A bibliometric study of research pertaining to the oldest-old (age eighty-five and older)
Source: J Med Libr Assoc. 2020 Jan 1;108(1):59–66. doi: 10.5195/jmla.2020.762 (PMC6919997; doi:10.5195/jmla.2020.762)
Supplement: Appendix A [file jmla-108-59-s001.pdf]

JMLA Journal of the Medical Library Association

SC
Supplemental content to
J Med Libr Assoc. Jan;108(1):dx.doi.org/10.5195/jmla.2020.762
www.jmla.mlanet.org
© Lund, Wang 2020

A bibliometric study of research pertaining to the oldest-old (age eighty-five and older)

Brady Daniel Lund; Ting Wang

APPENDIX A

Bibliometric studies related to aging and the elderly (1991–2019)

Alves KL, Silva AO, de Freitas Jordão do Amaral AK, de Moura SG, Evangelista CB, Pires de Sa CMC, Brenna S, do Céu Mendes Pinto Marques M, de Almeida JLT, Vitoriano MVTC, Moreira MASP. Theory of social representations and the aging: a bibliometric profile. Int Arch Med. 2016 Jul;9(147):1–9.

Ang HM, Kwan YH. Bibliometric analysis of journals in the field of geriatrics and gerontology. Geriatr Gerontol Int. 2017 Feb;17(2):357–60.

Antunez MY, Henry M. Where is research reported in gerontological nursing? an analysis to identify high impact journals, 1996–2013 [Internet]. 2015 [cited 21 May 2019]. <https://library.csun.edu/sites/default/files/users/mhenry/poster_inane_2014flarge.pdf>.

Antunez MY, Henry M. A comparative review of gerontological nursing citation data. Health Inf Libr J. 2016 Dec;33(4):257–68.

Asghar I, Cang S, Yu H. Assistive technology for people with dementia: an overview and bibliometric study. Health Inf Libr J. 2017 Mar;34(1):5–19.

Bergman EML. Social gerontology—integrative and territorial aspects: a citation analysis of subject scatter and database coverage. Behav Soc Sci Libr. 2011;30(3):154–75.

Boote J, Wong R, Booth A. Talking the talk or walking the walk? a bibliometric review of the literature on public involvement in health research published between 1995 and 2009. Health Expect. 2015 Feb;18(1):44–57.

Brown T, Gutman SA, Ho Y, Fong KNK. A bibliometric analysis of occupational therapy publications. Scandinavian J Occup Ther. 2018 Jan;25(1):1–14.

Chen H, Wan Y, Jiang S, Cheng Y. Alzheimer's disease research in the future: bibliometric analysis of cholinesterase inhibitors from 1993–2012. Scientometr. 2014 Mar;98(3):1865–77.

Conklin AI, Maguire ER, Monsivais P. Economic determinants of diet in older adults: systematic review. J Epidemiol Community Health. 2013 Sep;67(9):721–7.

da Silva EC, de Lima CLJ, Zaccara AAL, Evangelista CB, de Lourdes Neves de Oliveira E, de Sousa ATO, de Fátima Oliveira Coutinho Silva M, de Fátima Geraldo da Costa S, de Sa Franca JR. The scientific production about the Human Caring Theory: a bibliometric study. Int Arch Med. 2017 Mar;10(79):1–10.

Dominko M, Verbic M. Subjective well-being among the elderly: a bibliometric analysis. Qual Quant. 2019 May;53(3):1187–207.

Duarte MCS, da Costa SFG, da Nóbrega Morais GS, de Sa França JRF, Fernandes MA, Lopes MEL. Scientific production on the elderly person undergoing palliative care: bibliometric study. Cuidado e Fundamental. 2015 Jul/Sep;7(3):3093–109.

Duplenko IK, Burchinskii SG. Interdisciplinary research in gerontology: citation analysis. Fiziol Zh. 1991 Jan–Feb;37(1):114–7.

Feizabadi M, Vaziri E. A scientometric study of dementia research in geriatrics and gerontology journals. J Biomed Health. 2017 May;2(4):260–9.

Glazier R, Fry J, Badley E. Arthritis and rheumatism are neglected health priorities: a bibliometric study. J Rheumatol. 2001 Apr;28(4):706–11.

JMLA Journal of the Medical Library Association

Supplemental content to
J Med Libr Assoc. Jan;108(1):dx.doi.org/10.5195/jmla.2020.762
www.jmla.mlanet.org
© Lund, Wang 2020

Gu YH, Bai JB, Chen XL, Wu WW, Liu XX, Tan XD. Healthy aging: a bibliometric analysis of the literature. Exper Gerontol. 2019 Feb;116:93–105.

Guido D, Morandi G, Palluzzi F, Borroni B. Telling the story of frontotemporal dementia by bibliometric analysis. J Alzheimers Dis. 2015;48(3):703–9.

Jerez-Roig J, Guedes MB, Gonçalves MBO, Dias e Silva JM, de Lima KC. Analysis of the scientific production of the Brazilian Journal of Geriatrics and Gerontology: a bibliometric review. Revista Brasileira de Geriatria e Gerontologia. 2014;17(3):1–13.

Jiao Z, Ying MA, Hui Z. Bibliometrics analysis for the research of social work in the field of the health of the elderly in China [Internet]. Chinese Rural Health Service Administration; 2016 [cited 20 May 2019]. <http://en.cnki.com.cn/Article_en/CJFDTOTAL-ZNWS201606007.htm>.

Kirilov I, Atzeni M, Perra A, Moro D, Carta MG. Active aging and elderly's quality of life: comparing the impact on literature of projects funded by the European Union and USA. Clin Pract Epidemiol Ment Health. 2018 Jan 31;14:1–5.

Li T, Ho YS, Li CY. Bibliometric analysis on global Parkinson's disease research trends during 1991–2006. Neurosci Lett. 2008 Aug 29;441(3):248–52.

Lidor R, Miller U, Rotstein A. Is research on aging and physical activity really increasing? a bibliometric analysis. J Aging Phys Act. 1999;7(2):182–95.

Müller AM, Ansari P, Ebrahim NA, Khoo S. Physical activity and aging research: a bibliometric analysis. J Aging Phys Act. 2016 Jul;24(3):476–83.

Nagarajan R, Teixeira AAC, Silva S. The impact of population aging on economic growth: a bibliometric survey. Singap Econ Rev. 2017 Jun;62(2):275–96.

Navarro A, Lynd FE. Where does research occur in geriatrics and gerontology? J Am Geri Soc. 2005 Jun;53(6):1058–63.

Rikkert MGM, Have HAM, Hoefnagels WHL. Informed consent in biomedical studies on aging: survey of four journals. BMJ. 1996 Nov 2;313(7065):1117.

Rosen D, Engel RJ, Hunsaker AE, Engel Y, Detlefsen EG, Reynolds CF. Just say know: an examination of substance use disorders among older adults in gerontological and substance abuse journals. Soc Work Pub Health. 2013;28(3/4):377–87.

Run-lian Z. Bibliometric analysis of the tourism for elderly people in China. J Langfang Teachers Coll [Internet]. 2013 [cited 14 May 2019]. <http://en.cnki.com.cn/Article_en/CJFDTotal-HZJS201301017.htm>.

Shen C, Nguyen DT, Hsu P. Bibliometric networks and analytics on gerontology research. Libr Hi Tech. 2019;37(1):88–100.

Theander SS, Gustafson L. Publications on dementia in MEDLINE 1974–2009: a quantitative bibliometric study. Int J Geri Psych. 2013 May;28(5):471–8.

Yang GY, Wang LQ, Ren J, Zhang Y, Li ML, Zhu YT, Luo J, Cheng YJ, Li WY, Wayne PM, Liu JP. Evidence base of clinical studies on tai chi. PLoS One. 2015 Mar 16;10(3):e0120655.

Yaseen M. Literature on Alzheimer's disease 2003–2004: a bibliometric study [dissertation] [Internet]. Aligarh Muslim University; 2005 [cited 20 May 2019]. <http://ir.amu.ac.in/8442/1/DS%203443.pdf>.

Yeung AWK, Goto TK, Leung WK. The changing landscape of neuroscience research, 2006–2015: a bibliometric study. Front Neurosci. 2017Mar 21;11:article 120.

Zhou B, Duan C, Shi J, Tan X, Yu P. Analysis of international literatures on geriatrics and gerontology in mainland of China. Chin J Geriatr. 2013;6:675–8.
