# Supplementary material for: A bibliometric study of research pertaining to the oldest-old (age eighty-five and older)
Source: J Med Libr Assoc. 2020 Jan 1;108(1):59–66. doi: 10.5195/jmla.2020.762 (PMC6919997; doi:10.5195/jmla.2020.762)
Supplement: Appendix B [file jmla-108-59-s002.pdf]

JMLA Journal of the Medical Library Association

SC
Supplemental content to
J Med Libr Assoc. Jan;108(1):dx.doi.org/10.5195/jmla.2020.762
www.jmla.mlanet.org
© Lund, Wang 2020

A bibliometric study of research pertaining to the oldest-old (age eighty-five and older)

Brady Daniel Lund; Ting Wang

APPENDIX B

Articles with more than 100 citations (1991–2019)

Rank	Article	Citations
1	Franceschi C, Bonafe M, Valensin S, Olivieri F, de Luca M, Ottaviani E, de Benedictis G. Inflamm-aging: an evolutionary perspective on immunoscience. Ann NY Acad Sci. 2000 Jun;908(1):244–54.	1,589
2	Schächter F, Faure-Delanef L, Guénot F, Rouger H, Froguel P, Lesueur-Ginot L, Cohen D. Genetic associations with human longevity at the APOE and ACE loci. Nat Genet. 1994 Jan;6(1):29.	813
3	Lamberts SW, van den Beld AW, van der Lely AJ. The endocrinology of aging. Science. 1997 Oct 17;278(5337):419–24.	633
4	Schneider EL, Guralnik, JM. The aging of America: impact on health care costs. JAMA. 1990 May 2;263(17):2335–40.	510
5	Baltes PB, Smith J. New frontiers in the future of aging: from successful aging of the young old to the dilemmas of the fourth age. Gerontol. 2003 Mar–Apr;49(2):123–35. DOI: http://dx.doi.org/10.1159/000067946.	508
6	Biagi E, Nylund L, Candela M, Ostan R, Bucci L, Pini E, Nikkïla J, Monti D, Satokari R, Franceschi C, Brigidi P, De Vos W. Through ageing, and beyond: gut microbiota and inflammatory status in seniors and centenarians. PLoS One. 2010 May 17;5(5): e10667.	475
7	Suh Y, Atzmon G, Cho MO, Hwang D, Liu B, Leahy DJ, Barzilai N, Cohen P. Functionally significant insulin-like growth factor I receptor mutations in centenarians. Proceed Natl Acad Sci USA. 2008 Mar 4;105(9):3438–42.	416
8	Longo VD, Finch CE. Evolutionary medicine: from dwarf model systems to healthy centenarians? Science. 2003 Feb 28;299(5611):1342–6.	405
9	Heyn H, Li N, Ferreira HJ, Moran S, Pisano DG, Gomez A, Diez J, Sanchez-Mut JV, Setien F, Carmona FJ, Puca AA, Sayols S, Pujana MA, Serra-Musach J, Iglesias-Platas I, Formiga F, Fernandez AF, Fraga MF, Heath SC, Valencia A, Gut IG, Wang J, Esteller M. Distinct DNA methylomes of newborns and centenarians. Proceed Natl Acad Sci USA. 2002 Jun 26;109(26):10522–7.	371
10	Blazer D, Burchett B, Service C, George LK. The association of age and depression among the elderly: an epidemiologic exploration. J Gerontol. 1991 Nov;46(6): M210–M215.	335
11	Sansoni P, Cossarizza A, Brianti V, Fagnoni F, Snelli G, Monti D, Marcato A, Passeri G, Ortolani C, Forti E. Lymphocyte subsets and natural killer cell activity in healthy old people and centenarians. Blood. 1993 Nov 1;82(9):2767–73.	326

SC
Supplemental content to
J Med Libr Assoc. Jan;108(1):dx.doi.org/10.5195/jmla.2020.762
www.jmla.mlanet.org
© Lund, Wang 2020

Rank	Article	Citations
12	Weverling-Rijnsburger AW, Blauw GJ, Lagaay AM, Knock DL, Meinders AE, Westendorp RG. Total cholesterol and risk of mortality in the oldest old. Lancet. 1999 Oct 18;350(9085):1119–23.	324
13	Bruunsgaard H, Andersen-Ranberg K, Jeune B, Pedersen AN, Skinhøj P, Pedersen BK. A high plasma concentration of TNF-α is associated with dementia in centenarians. J Gerontol Series A. 1999 Jul;54(7):M357–M364.	304
14	Harris T, Kovar MG, Suzman R, Kleinman JC, Feldman JJ. Longitudinal study of physical ability in the oldest-old. Am J Pub Health. 1989 Jun;79(6):698–702. DOI: http://dx.doi.org/10.2105/AJPH.79.6.698.	298
15	Vasto S, Candore G, Balistreri CR, Caruso M, Colonna-Romano G, Grimaldi MP, Listi F, Nuzzo D, Lio D, Caruso C. Inflammatory networks in ageing, age-related diseases and longevity. Mech Aging Dev. 2007 Jan;128(1):83–91.	297
16	Byers AL, Yaffe K, Covinsky KE, Friedman MB, Bruce ML. High occurrence of mood and anxiety disorders among older adults: the National Comorbidity Survey Replication. Arch Gen Psych. 2010 May;67(5):489–96.	265
17	Evert J, Lawler E, Bogan H, Perls T. Morbidity profiles of centenarians: survivors, delayers, and escapers. J Gerontol A Biol Sci Med Sci. 2003 Mar;58(3): 232–7.	249
18	Nygren B, Aléx L, Jonsén E, Gustafson Y, Norberg A, Lundman B. Resilience, sense of coherence, purpose in life and self-transcendence in relation to perceived physical and mental health among the oldest old. Aging Ment Health. 2005 Jul;9(4):354–62.	228
19	Perls TT, Wilmoth J, Levenson R, Drinkwater M, Cohen M, Bogan H, Joyce E, Brewster S, Kunkel L, Puca A. Life-long sustained mortality advantage of siblings of centenarians. Proc Natl Acad Sci USA. 2002 Jun 11;99(12):8442–7.	224
19	von Faber M, Bootsma-van der Wiel A, van Exel E, Gussekloo J, Lagaay AM, van Dongen E, Knook DL, van der Geest S, Westendorp RG. Successful aging in the oldest old: who can be characterized as successfully aged? Archives Int Med. 2001 Dec 10–24;161(22):2694–700.	224
21	Franceschi C, Bonafè M. Centenarians as a model for healthy aging. Biochem Soc Trans. 2003 Apr;31(2):457–61.	201
21	Franceschi C, Valensin S, Bonafe M, Paolisso G, Yashin AI, Monti D, De Benedictis G. The network and the remodeling theories of aging: historical background and new perspectives. Exper Gerontol. 2000 Sep;35(6–7): 879–96.	201
23	Taekema DG, Gussekloo J, Maier AB, Westendorp RG, de Craen AJ. Handgrip strength as a predictor of functional, psychological and social health. a prospective population-based study among the oldest old. Age Aging. 2010 May;39(3):331–7.	198

Supplemental content to
J Med Libr Assoc. Jan;108(1):dx.doi.org/10.5195/jmla.2020.762
www.jmla.mlanet.org
© Lund, Wang 2020

Rank	Article	Citations
24	Franceschi C, Motta L, Valensin S, Rapisarda R, Franzone A, Berardelli M, Motta M, Monti D, Bonafè M, Ferrucci L, Deiana L, Pes GM, Carru C, Desole MS, Barbi C, Sartoni G, Gemelli C, Lescai F, Olivieri F, Marchegiani F, Cardelli M, Cavallone L, Gueresi P, Cossarizza A, Troiano L, Pini G, Sansoni P, Passeri G, Lisa R, Spazzafumo L, Amadio L, Giunta S, Stecconi R, Morresi R, Viticchi C, Mattace R, De Benedictis G, Baggio G. Do men and women follow different trajectories to reach extreme longevity? Italian Multicenter Study on Centenarians (IMUSCE). Aging (Milano). 2000 Apr;12(2):77–84.	186
25	de Craen AJ, Heeren TJ, Gussekloo J. Accuracy of the 15-item geriatric depression scale (GDS-15) in a community sample of the oldest old. Int J Geriatr Psychiatry. 2003 Jan;18(1):63–6.	181
26	Longo VD, Antebi A, Bartke A, Barzilai N, Brown-Borg HM, Caruso C, Curiel TJ, de Cabo R, Franceschi C, Gems D, Ingram DK, Johnson TE, Kennedy BK, Kenyon C, Klein S, Kopchick JJ, Lepperdinger G, Madeo F, Mirisola MG, Mitchell JR, Passarino G, Rudolph KL, Sedivy JM, Shadel GS, Sinclair DA, Spindler SR, Suh Y, Vijg J, Vinciguerra M, Fontana L. Interventions to slow aging in humans: are we ready? Aging Cell. 2015 Aug;14(4):497–510.	180
27	Corrada MM, Brookmeyer R, Paganini-Hill A, Berlau D, Kawas CH. Dementia incidence continues to increase with age in the oldest old: the 90+ study. Ann Neuro. 2010 Jan;67(1):114–21.	176
28	Mari D, Mannucci PM, Coppola R, Bottasso B, Bauer KA, Rosenberg RD. Hypercoagulability in centenarians: the paradox of successful aging. Blood. 1995 Jun 1;85(11):3144–9.	167
29	Andersen-Ranberg K, Schroll M, Jeune B. Healthy centenarians do not exist, but autonomous centenarians do: a population-based study of morbidity among Danish centenarians. J Am Geri Soc. 2001 Jul;49(7):900–8.	166
29	Kagawa Y. Impact of Westernization on the nutrition of Japanese: changes in physique, cancer, longevity and centenarians. Prev Med. 1978;7(2):205–17.	166
31	Hitt R, Young-Xu Y, Silver M, Perls T. Centenarians: the older you get, the healthier you have been. Lancet. 1999 Aug 21;354(9179):652.	157
32	Coppola R, Mari D, Lattuada A, Franceschi C. Von Willebrand factor in Italian centenarians. Haematologica. 2003 Jan;88(1):39–43.	147
33	Camicioli R, Howieson D, Oken B, Sexton G, Kaye J. Motor slowing precedes cognitive impairment in the oldest old. Neuro. 1998 May;50(5):1496–8.	146
34	Mariotti S, Barbesino G, Caturegli P, Bartalena L, Sansoni P, Fagnoni F, Monti D, Fagiolo U, Franceschi C, Pinchera A. Complex alteration of thyroid function in healthy centenarians. J Clin Endocrinol Metab. 1993 Nov;77(5):1130–4.	142
34	Perls TT, Bubrick E, Wager CG, Vijg J, Kruglyak L. Siblings of centenarians live longer. Lancet. 1998 May 23;351(9115):1560.	142

SC
Supplemental content to
J Med Libr Assoc. Jan;108(1):dx.doi.org/10.5195/jmla.2020.762
www.jmla.mlanet.org
© Lund, Wang 2020

Rank	Article	Citations
36	Caruso C, Lio D, Cavallone L, Franceschi C. Aging, longevity, inflammation, and cancer. Ann NY Acad Sci. 2004 Dec;1028:1–13.	139
37	Dunlop DD, Manheim LM, Song J, Chang RW. Gender and ethnic/racial disparities in health care utilization among older adults. J Gerontol B Psychol Sci Soc Sci. 2002 Jul;57(4):S221–S233.	134
38	Atzmon G, Cho M, Cawthon RM, Budagov T, Katz M, Yang X, Siegel G, Bergman A, Huffman DM, Schechter CB, Wright WE, Shay JW, Barzilai N, Govindaraju DR, Suh Y. Evolution in health and medicine Sackler colloquium: genetic variation in human telomerase is associated with telomere length in Ashkenazi centenarians. Proc Natl Acad Sci USA. 2010 Jan 26;107(suppl 1):1710–7.	133
39	Soldo BJ, Manton KG. Changes in the health status and service needs of the oldest old: current patterns and future trends. Milbank Meml Fund Q. 1985 Spring;63(2):286–319. DOI: http://dx.doi.org/10.2307/3349882.	130
40	Atzmon G, Barzilai N, Hollowell JG, Surks MI, Gabriely I. Extreme longevity is associated with increased serum thyrotropin. J Clinic Endocrin Metabol. 2009 Apr;94(4):1251–4.	129
40	Lennartsson C, Silverstein M. Does engagement with life enhance survival of elderly people in Sweden? the role of social and leisure activities. J Gerontol B Psychol Sci Soc Sci. 2001 Nov;56(6):S335–S342.	129
42	Andersen SL, Sebastiani P, Dworkis DA, Feldman L, Perls TT. Health span approximates life span among many supercentenarians: compression of morbidity at the approximate limit of life span. J Gerontol A Biol Sci Med Sci. 2012 Apr;67(4):395–405.	128
43	Atzmon G, Schechter C, Greiner W, Davidson D, Rennert G, Barzilai N. Clinical phenotype of families with longevity. J Am Geriatr Soc. 2004 Feb;52(2):274–7.	126
43	Horgas AL, Wilms HU, Baltes MM. Daily life in very old age: everyday activities as expression of successful living. Gerontologist. 1998 Oct;38(5):556–68.	126
45	Mehta KM, Yaffe K, Covinsky KE. Cognitive impairment, depressive symptoms, and functional decline in older people. J Am Geriatr Soc. 2002 Jun;50(6):1045–50.	123
46	Chondrogianni N, Petropoulos I, Franceschi C, Friguet B, Gonos ES. Fibroblast cultures from healthy centenarians have an active proteasome. Exper Geront. 2000 Sep;35(6–7):721–8.	121
46	van den Berg E, Biessels GJ, de Craen AJM, Gussekloo J, Westendorp RGJ. The metabolic syndrome is associated with decelerated cognitive decline in the oldest old. Neurology. 2007 Sep 4;69(10):979–85.	121
48	Carey EC, Walter LC, Lindquist K, Covinsky KE. Development and validation of a functional morbidity index to predict mortality in community-dwelling elders. J Gen Int Med. 2004 Oct;19(10):1027–33.	120
49	Himes CL. Obesity, disease, and functional limitation in later life. Demography. 2003 Feb;37(1):73–82.	118

SC
Supplemental content to
J Med Libr Assoc. Jan;108(1):dx.doi.org/10.5195/jmla.2020.762
www.jmla.mlanet.org
© Lund, Wang 2020

Rank	Article	Citations
50	Corrada MM, Brookmeyer R, Berlau D, Paganini-Hill A, Kawas CH. Prevalence of dementia after age 90: results from the 90+ study. Neurology. 2008 Jul 29;71(5):337–43.	117
51	Gilleard C, Higgs P. Aging without agency: theorizing the fourth age. Aging Ment Heal. 2010 Mar;14(2):121–8.	116
52	Reed PG. Self-transcendence and mental health in oldest-old adults. Nurs Res. 1991 Jan–Feb;40(1):5–11.	114
53	Kaye JA, Oken BS, Howieson DB, Howieson J, Holm LA, Dennison K. Neurologic evaluation of the optimally healthy oldest old. Archives Neuro. 1994 Dec;51(12):1205–11.	113
54	Fiori KL, Smith J, Antonucci TC. Social network types among older adults: a multidimensional approach. J Gerontol B Psychol Sci Soc Sci. 2007 Nov;62(6):P322–P330.	111
55	Boonen S, McClung MR, Eastell R, El-Hajj Fuleihan G, Barton IP, Delmas P. Safety and efficacy of risedronate in reducing fracture risk in osteoporotic women aged 80 and older: implications for the use of antiresorptive agents in the old and oldest old. J Am Geriatr Soc. 2004 Nov;52(11):1832–9.	109
56	Gondo Y, Hirose N, Arai Y, Inagaki H, Masui Y, Yamamura K, Shimizu K, Takayama M, Ebihara Y, Nakazawa S, Kitagawa K. Functional status of centenarians in Tokyo, Japan: developing better phenotypes of exceptional longevity. J Gerontol A Biol Sci Med Sci. 2006 Mar;61(3):305–10.	107
56	Tschanz JT, Corcoran C, Skoog I, Khachaturian AS, Herrick J, Hayden KM, Welsh-Bohmer KA, Calvert T, Norton MC, Zandi P, Breitner JC; Cache County Study Group. Dementia: the leading predictor of death in a defined elderly population: the Cache County Study. Neurology. 2004 Apr 13;62(7):1156–62.	107
58	Maier H, Smith J. Psychological predictors of mortality in old age. J Gerontol B Psychol Sci Soc Sci. 1999 Jan;54(1):P44–P54.	106
59	Marini C, Baldassarre M, Russo T, De Santis F, Sacco S, Ciancarelli I, Carolei A. Burden of first-ever ischemic stroke in the oldest old: evidence from a population-based study. Neurology. 2004 Jan 13;62(1): 77–81.	105
60	Oates DJ, Berlowitz DR, Glickman ME, Silliman RA, Borzecki, AM. Blood pressure and survival in the oldest old. J Am Geriatr Soc. 2007 Mar;55(3):383–8.	104
61	Jopp D, Rott, C. Adaptation in very old age: exploring the role of resources, beliefs, and attitudes for centenarians' happiness. Psych Aging. 2006 Jul;21(2):266–80.	102
62	Baert V, Gorus E, Mets T, Geerts C, Bautmans I. Motivators and barriers for physical activity in the oldest old: a systematic review. Aging Res Rev. 2011 Sep;10(4):464–74.	100
